# Depressive symptoms and marital adjustment among primary care patients with erectile dysfunction in Umuahia, Nigeria

**DOI:** 10.4102/sajpsychiatry.v22i1.979

**Published:** 2016-08-19

**Authors:** Nwaonu C. Nwakanma, John N. Ofoedu

**Affiliations:** 1 Department of Psychiatry, Federal Medical Centre, Umuahia, Nigeria; 2 Department of Family Medicine, Federal Medical Centre, Umuahia, Nigeria

## Abstract

**Objectives:**

The aim of this study was to investigate the relationship between erectile dysfunction (ED), marital adjustment and depression.

**Methods:**

The survey was conducted among primary care patients at Federal Medical Centre, Umuahia. Subjects were 678 married, male primary care patients; aged 20–70 years (mean age = 45 years). ED was assessed by International Index of Erectile Function 5 (IIEF-5) score, the presence of clinically significant depressive symptoms was assessed with the 5-item version of the Center for Epidemiological Studies Depression Scale (CES-D), and marital adjustment was assessed with the Revised Dyadic Adjustment Scale (RDAS).

**Results:**

The prevalence of probable depression by CES-D and ED by IIEF-5 score was 20.9% and 26.0%, respectively. Marital distress was rampant (62.0%) among subjects with ED (*p* < 0.05, χ^2^ = 196.58). Erectile dysfunction was associated with marital adjustment (*p* < 0.05). Partial correlation revealed that depression affects both ED and marital adjustment, and is closely related to both variables.

**Conclusion:**

Partner involvement and screening for depression should be emphasised in the care of patients with ED.

## Introduction

Erectile dysfunction (ED) is the most common sexual problem in men. It often causes serious distress and has a profound effect on intimate relationships, quality of life and overall self-esteem.^1,2^ It was defined in 1993 by the US National Institute of Health Consensus Development panel on impotence as the inability to achieve and/or maintain an erection sufficient for satisfactory sexual intercourse.^2^ In Nigeria, it is estimated that one out of every two men has ED. According to various local studies, the prevalence rate is between 19.8% and 57.4% for Nigerian men.^3,4,5,6,7,8^

Depression has been documented as a recurring morbidity among ED patients in other regions of the world, while marriage is a major source of social support and a predictor of health.^8,9,10,11,12,13,14^ The impact of several medical conditions on marital satisfaction has been documented.^8,9^ Unfortunately, even though ED affects an essential ingredient of marital relationships, the association between ED, marital satisfaction and depression has been scarcely explored, especially in sub-Saharan Africa. This gap in knowledge underscores unmet needs for consultation-liaison psychiatry services in the management of ED patients in our locale.

This study was therefore designed to describe the prevalence and pattern of ED, and its relationship with marital satisfaction and depression. It was carried out among adult male patients receiving care at the general outpatient clinic of the Federal Medical Centre, Umuahia, Nigeria.

## Methods and materials

### Study location

Umuahia is a town located in Abia state, South-Eastern Nigeria. It was a colonial railway hub, a suburb intended for the gathering of local agricultural produce. It is currently the Capital of Abia state. The town is densely populated by indigenous ethnic Igbos. The inhabitants of the town are mostly civil servants, petty traders, artisans and farmers. The scale of economic and social activities in Umuahia is low compared with the prime industrial and commercial cities in Nigeria, such as Lagos and Port Harcourt. Umuahia is about 2 h drive from the two major commercial cities in the South-Eastern region of Nigeria, namely Onitsha and Aba.

### Study site

This study was conducted at Federal Medical Centre, Umuahia (formerly Queen Elizabeth Hospital). This hospital was founded as a missionary hospital and commissioned in 1956 by Sir Clement Pleas, on behalf of Queen Elizabeth. It had undergone successive acquisition and conversions by the state and federal governments. Initially, it was upgraded to a state general hospital. Currently, it is a 375 bedded federally owned tertiary hospital, with about 76 full-time specialist consultants and 105 post-graduate resident doctors in the various medical specialities. The tripartite mandate of the hospital is service delivery, training and research. It serves as a referral centre for primary and secondary public health institutions as well as missionary and private hospitals in Abia state and neighbouring communities.

The hospital runs a primary care clinic within the tertiary hospital setting of the medical centre. All adult patients excluding those who need emergency health care services, paediatric patients and antenatal women are first seen at this family medicine clinic/general outpatient’s clinic where diagnoses are made. Patients who need primary care are managed and followed up in the clinic, while those who need specialist/specialised care are referred to the respective specialist clinics for further management. The clinic is run by consultant family physicians and post-graduate resident medical doctors. The family medicine clinic runs between 8 am to 4 pm daily, from Monday to Friday. The clinic sees between 53 and 70 patients on every clinic day. There is an approximately equal gender mix (male to female ratio) in the patient population.

### Study procedure

A cross-sectional survey of 678 married adult male patients who presented at the general outpatients clinic, Federal Medical Centre, Umuahia, was carried out over a 3-month period. About 13 patients were recruited on each clinic day. Only married adult males who have been living with their partners for at least 2 years were recruited.

Recruitment was by systematic random sampling. We sampled every third married male that presented at the nurses’ station for a specific clinic operation (measurement of vital signs). The first subject on the ‘skip of 3’ sample frame was picked by ballot method, out of the first three eligible and consenting patients registered for the clinic operation. This was done on each clinic day until the sample size was completed. The number 3 was gotten by dividing the study population by the estimated sample size. A written consent was obtained from each subject, after they were informed about the nature, extent and purpose of the research. Ethical clearance was obtained from the ethical committee of the hospital. Subjects with ED and probable depression received a brief counselling and were then referred to our specialist psychiatry clinic for review and expert management.

The minimum sample size was calculated using the formula:

$$N=\frac{Z^{2}\text{PQ}}{D^{2}}$$ [Eqn 1]

The prevalence rate of ED among male patients in primary care settings estimated at 57.4% by Shaeer et al. was used.^3^ Minimum sample size (at 80% anticipated response rate) was calculated to be 470.

However, a total of 806 married adult males were randomly selected to participate in the study. After completing the socio-demographic questionnaire, the 5-item version of the Center for Epidemiologic Studies Depression Scale (CES–D) questionnaire and the International Index of Erectile Function (IIEF-5) questionnaires, all the selected subjects were requested to return for follow-up in 1 week with their spouse. During this follow-up visit, the Revised Dyadic Adjustment Scale (RDAS) was completed by the couple. The actual response rate was 85.7 (691/806). Furthermore, a small number of patients (*n* = 13) were excluded from the data analysis owing to missing values in the variables considered. Therefore, the final sample size was 678.

### Study instruments

The survey instruments were:

1. Socio-demographic questionnaire: The socio-demographic information was collected by self-report. Patients’ medical diagnosis made by the attending family physician was also recorded.
2. Center for Epidemiologic Studies Depression Scale Questionnaire: Current depressive symptoms were assessed using the shortened (5-item) CES-D, with scores ranging from 0 to 15 and higher scores denoting more depressive symptoms.^15^ CES-D 5-item version has good sensitivity (> 0.84), specificity (≥ 0.80) and high validity (> 0.90) for all identified cut-points in identifying patients classified as depressed by the full 20-item scale.^15^
  This study adopted the standard CES-D ‘0–1–2–3’ response format, and by means of Kohout’s equivalence formula, the cut-point score was set at 4.^16,17^ Patients were classified into those with ‘probable depression’ (5-item CES-D score of ≥ 4) and ‘not depressed’ (5-item CES-D score of < 4). The CES-D has been validated in Nigeria, the 5-item version CES-D was used in the cross-national study conducted by Shaeer et al. to evaluate Nigerian subjects.^3^
3. The International Index of Erectile Function (IIEF-5) Questionnaire: Presence of ED was defined as IIEF-5 score < 21. Severity of ED is further categorised into score 5–10, severe ED; score 11–15, moderate ED; score 16–20, mild ED; and score 21–25, normal (no ED).^18^
4. Revised Dyadic Adjustment Scale: Marital adjustment was assessed using the RDAS.^19,20^ The RDAS consists of 14 items evaluating the couple’s agreement on decisions and appropriate behaviour, marital satisfaction and marital cohesion. The RDAS scores range between 0 and 69, with a low score indicating a distressed dyadic adjustment. The RDAS provides a total score (RDAS-T). The patients were categorised according to the total score of 48 as a cut-off point so as to distinguish the maritally distressed (RDAS < 48) from the non-distressed ones (RDAS ≥ 48).^19,20^

### Data analysis

Statistical analysis was performed using the Statistical Package for Social Sciences (SPSS Version 17.0 for Windows, SPSS Incorporated, Chicago, IL, USA). Confidence interval will be set to 95% and *p*-value of less than 0.05 was considered significant. Partial correlation technique was used to examine the relationship between the study variables.

### Results

A total of 678 male patients, aged 20–70 years (*M* = 45, SD = 12.1), were reviewed at the general outpatient clinic of our hospital. Of the subjects, 176 (*n* = 176; 26%) had ED. Table 1 shows the prevalence of ED across the various age groups. Subjects within the 20- to 30-year age group had a low prevalence of ED (9.0%). The prevalence of ED among all the subjects showed a bimodal peak of 33.3%, which occurred in subjects aged 31–40 years and 51–60 years.

**TABLE 1:** Prevalence of erectile dysfunction across the age distribution of the subjects.

Years	ED present	ED absent	Total	%
		
	Frequency (%)	Frequency (%)	Frequency	
20–30	8 (9.0)	80 (91.0)	88	13.0
31–40	43 (33.3)	86 (67.7)	129	19.0
41–50	57 (23.4)	187 (76.6)	44	36.0
51–60	35 (33.3)	70 (67.7)	105	15.5
61–70	33 (29.5)	79 (70.5)	112	16.5

**Total**	**176 (26)**	**502 (74)**	**678**	**100**

ED, erectile dysfunction.

Table 2 presents the pattern and prevalence of probable depression among the subjects. A 2 x 2 *χ*^2^ test was conducted, and the analysis was found to be statistically significant. The observed frequencies for the cells can be found in Table 2. Of all the subjects reviewed in this study, 142 (*n* = 142; 20.9%) had probable clinical depression. The prevalence of probable depression among subjects with ED was 42.6%, compared with 13.3% among subjects without ED (*p* < 0.05, *χ*^2^ = 67.41).

**TABLE 2:** Depressive features and marital adjustment among the subjects.

Variable	ED present *n* (%)	ED absent *n* (%)	Total *n* (%)	Chi square
**Depressive features (by 5-item CES-D)**				
Probable depression	75 (42.6)	67 (13.3)	142 (20.9)	67.414
No depression	101(57.4)	435 (86.7)	536 (79.1)	*p* < 0.05
**Marital adjustment**				
Distressed	126 (71.6)	77 (15.3)	203 (30.0)	196.5787
Non-distressed	50 (28.4)	425 (84.7)	475 (70.0)	*p* < 0.05

**Total**	**176 (100.0)**	**502 (100.0)**	**678 (100.0)**	

ED, erectile dysfunction.

Table 2 also shows the pattern and prevalence of marital adjustment among the subjects. The overall prevalence of marital distress was 30%. Marital distress was rampant (71.6%) among subjects with ED, compared with those without ED (15.3%). The 2 x 2 *χ*^2^ test conducted to assess the equal distribution of marital adjustment across subjects with ED and those without ED was found to be statistically significant (*p* < 0.05, *χ*^2^ = 196.58).

**FIGURE 1:**
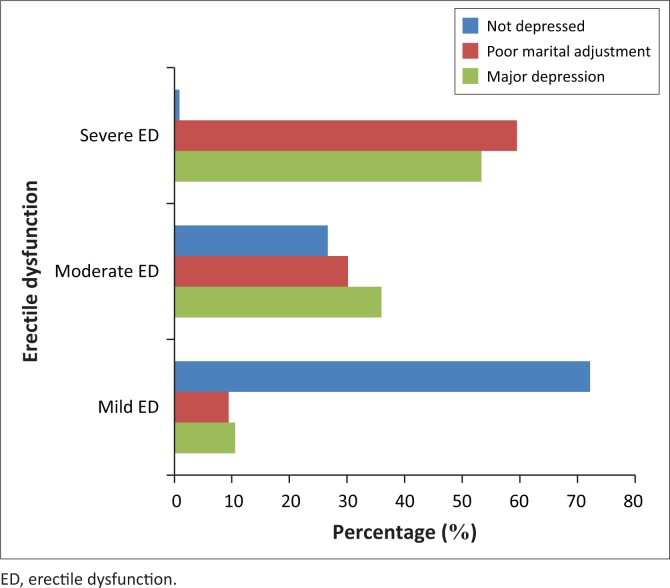
Distribution of depressive features and marital adjustments among subjects with erectile dysfunction.

Figure 1 is a bar chart that depicts the distribution of probable clinical depression and marital adjustment among the subjects with ED. It shows an increase in the prevalence of probable depression and poor marital adjustment among the subjects, occurring in tandem with increasing severity of ED. Clinically significant depressive features occurred at a higher frequency (*n* = 40; 53.33%) among subjects with severe ED, compared with those with mild (*n* = 8; 10.67%) and moderate (*n* = 27; 36.00%) forms of ED. Conversely, only one (*n* = 1; 0.99%) of the subjects with ‘ED and no depression’ was of the severe ED category, while 73 (72.28%) of them had mild ED.

ED, erectile dysfunction.

It is also noteworthy that subjects with severe ED had a higher prevalence of poor marital adjustment (*n* = 75; 59.52%), in comparison to those with mild (*n* = 12; 9.52%) and moderate (*n* = 38; 30.16%) forms of ED. This is also illustrated in Figure 1.

Spearman correlation was computed to explore the relationship between ED and marital adjustment. This analysis was found to be statistically significant, *r* (176) = 0.568, *p* < 0.05, indicating a strong positive relationship between ED and marital adjustment. The relationship was then subjected to a first-order partial correlation in order to explore the relationship while controlling for the effects of depression. The first-order correlation was found to be statistically significant, *r* (126) = 0.542, *p* < 0.05, indicating that a relationship between ED and marital adjustment exists above and beyond the effects of probable depression. Nevertheless, the strength of the relationship between ED and marital adjustment was lessened, while the effect of probable depression was controlled, that is, the presence of clinically significant depressive features affects both ED and marital adjustment and is closely related to both variables.

## Discussion

The prevalence of ED found among male patients (aged 20 to 70 years) attending our primary care (26%) is low when compared with similar studies conducted in other regions of Nigeria. For example, Idung et al. and Fatusi et al. reviewed primary care patients (aged 20 to 70 years) in the Niger Delta region^7^ and South-West^5^ region of Nigeria, and reported ED prevalence rates of 41.5% and 43.6%, respectively. Shaeer et al. reviewed random samples of 984 men (aged 35 to 70 years) attending clinics in Ibadan, Kano, Enugu and Lagos and reported a national prevalence of 57.4%.^3,10^ Among subjects in our study within that age range only 27.1% had ED.

It can therefore be surmised that the prevalence of ED among patients attending our primary care clinic is below the national average and below the prevalence in other regions of Nigeria. It is noteworthy that all of our subjects were ethnic Igbos of South-East Nigeria, among whom manhood and masculinity is strongly associated with potency and physical virility.^21,22^ Perhaps, such ethno-culturally determined factors colour the perception of ED and results in under-reporting of symptoms of ED among this unique cultural subset. This assertion is somewhat corroborated by the inter-ethnic prevalence of ED reported by Oladiji, Kayode and Parakoyi.^6^ Their study documented a high overall ED prevalence of 46.9% among a community dwelling cosmopolitan population that comprised the local Yoruba population, Igbo migrants and Hausa settlers in Ilorin, Nigeria. However, when they analysed the prevalence rate of ED among the various ethnic groups, they found a lower prevalence of ED among the Igbo respondents, with 64.9% of the Igbo respondents reporting ‘no ED’, compared with the Yoruba (50.5%) and Hausas (43.8%) respondents.^6^

Nigeria is an ethno-culturally diverse nation, and subtle differences exist in the culturally sanctioned perceptions and causal attributions of ED endorsed by the subjects across ethnic divides.^6,21,22^ Cultural variations in the prevalence of ED have been documented by previous studies in Europe and Asia. For example, Lewis^23^ analysed 10 studies investigating the prevalence of ED in Asian countries and observed that these countries have higher rates of ED than comparable European populations. Similarly, Nicosi et al.^24^ and Ho^25^ demonstrated large variation in the prevalence rate of ED between Asian countries. Consequently, in their treatise, Vishal and Bhugra surmised that sexual dysfunction was culturally influenced, as cultures create and colour myths about sexuality, masculinity and sexual prowess and behaviour.^22^

Subjects with ED in our study also had a higher prevalence of depressive features, compared with a previous study conducted by Okulate et al.^11^ in a Centre in South-West Nigeria. Okulate et al.^11^ reported a 10% prevalence of depression among ED patients in a primary care setting. On the contrary, and in comparison, ED patients in our centre were four times more likely to be depressed compared with their counterparts in the study conducted in South-West Nigeria. Perhaps, culturally influenced perceptions, causal attributions and social cognitions about ED would also have a significant effect on the prevalence of depressive features among ED patients. Further epidemiological interrogation of these observed variations would require a multicentre study conducted across the various regions of Nigeria.

It is conceivable that the association we found between ED, marital satisfaction and probable depression is a consequence of the negative effect of ED on quality of marital relationship. The marital impact of ED has previously been documented by Avasthi et al.^26^ in India. In their study, Avasthi et al. found that spouses of men with ED have significantly lower levels of marital and sexual satisfaction and higher levels of psychiatric symptoms than the controls. Furthermore, epidemiological studies have in the past associated men who are separated or divorced with a higher prevalence of ED, compared with those who remained married or living with a partner.^6^

Previous studies have suggested that ED has a direct impact on marital satisfaction, affects partner self-esteem and can cascade into the couple alienating themselves emotionally and physically. Ultimately, ED is believed to be involved in one in five failed marriages.^6,26,27^

Marital satisfaction is related to the level and quality of general health, life satisfaction and loneliness sense, and reduces mortality risk. Laboratory-based studies and community surveys have documented the impact of marital quality on cardiovascular and hormonal responses, as well as on the incidence and prognosis of physical diseases and psychological disorders.^28,29,30^

Conversely, this association could also imply that marital problems predated ED and were a contributory factor in the development of ED and depression among the subjects.^1,27^ Notwithstanding the causal direction of relationship, this study surmises that partner involvement and relationship quality are critical ingredients in the care of ED patients. Previous studies, including the multinational Men’s Attitudes to Life Events and Sexuality (MALES) study, have also highlighted the importance of relationship quality and partner involvement in the long-term management of ED, unless the informed patient is unwilling.^29,30,31^

Secondly, early detection and appropriate use of culturally sensitive interventions targeting depression among ED patients could also minimise negative marital outcome for affected patients. We therefore concur with Okulate et al. on the utility of simple questionnaires in primary care settings.^10^ Such questionnaires, if applied routinely, would increase the detection rate of ED and comorbid depression, and thereby engender early intervention. Psychological interventions for depression in patients with ED should also be a focal point for marital interventions.^28,32^ Among this patient group, the utility of the antidepressant medication which rarely causes sexual side effects (bupropion) cannot be over emphasised.^1,27,32,33,34.^

Our finding also highlights the need for epidemiological studies that will further elucidate the relationship between ED and marital distress in Nigeria. Future research should also explore the under-reporting of ED among primary care patients in various regions of Nigeria and the proximate determinants of that pattern of health-seeking behaviour. There is also a need for public enlightenment campaigns on ED to reduce sexual myths, stigma and cultural barriers to orthodox care of ED patients in South-East Nigeria.

### Limitations

As a cross-sectional questionnaire survey, data collection was limited to symptom endorsement and self-report. The study was clinic-based, conducted in one city and in one tertiary centre. It was not a population-based epidemiologic study. Nonetheless, we believe that our sample size, sampling method and high response rate would have minimised any existing bias.

## Conclusion

This study established a statistical correlation between ED, probable depression and relationship quality in a unique cultural subset. It also revealed that the prevalence of probable depression and poor marital adjustment among ED patients increases in tandem with increasing severity of ED. It also provided a modest ground to infer that there are culturally influenced regional differences in the primary care prevalence of ED in Nigeria. The study also highlights the marital impact of ED and points the direction for future epidemiological research. Partner involvement and screening for depression should be emphasised in the care of patients with ED.
